# A Curious Case of Weakness: Antineutrophil Cytoplasmic Antibody-Associated Vasculitis Presenting With Muscle Weakness

**DOI:** 10.7759/cureus.63453

**Published:** 2024-06-29

**Authors:** Okechukwu C Okoye, Sunita Paudyal, Shannon E Iriza, Jon Wilson

**Affiliations:** 1 Rheumatology, Prisma Health, University of South Carolina (USC) Rheumatology, Columbia, USA; 2 Pathology, Arkana Laboratories, Little Rock, USA

**Keywords:** symmetrical muscle edema, mpo-anca vasculitis, bilateral limb weakness, inflammatory myositis, antineutrophil cytoplasmic antibody (anca) associated vasculitis (aav)

## Abstract

Disease manifestations of antineutrophil cytoplasmic antibody (ANCA)-associated vasculitis (AAV), a small vessel vasculitis with multisystemic effects, include respiratory, renal, nervous, gastrointestinal, and skin implications. Muscle weakness and inflammatory myopathy are rare manifestations of AAV. We report the case of a 77-year-old female with a medical history of hypothyroidism and osteoarthritis who presented with a two-month history of worsening muscle weakness (mainly proximal). She endorsed dysphagia, a 40-lb unintentional weight loss, and persistent sinusitis with middle ear effusions, requiring bilateral tympanostomy. The physical examination was notable for 2/5 muscle strength in her hip flexors and extensors, with 4/5 strength in other extremities. Lower extremity MRI showed diffuse intramuscular edema between fat planes and intramuscular septal regions. Erythrocyte sedimentation rate (70 mm/hr), C-reactive protein (141 mg/L), creatine kinase (690 U/L), and anti-myeloperoxidase (MPO) antibodies (>999 AU/mL) were elevated. A thigh biopsy revealed fibrinoid necrosis of small intramuscular arteries, confluent circumferential granulomatous vessel wall inflammation, and associated mild chronic inflammation, including occasional eosinophils and a few plasma cells. She was diagnosed with MPO-positive AAV. The patient was started on high-dose steroids (prednisone), with a taper on a disease-modifying agent, azathioprine, with significant improvement in symptoms over the next four months and complete resolution at 16-month follow-up. This patient’s clinical presentation of predominant lower extremity weakness due to inflammatory myositis is an unusual manifestation of AAV. Clinicians should keep a broad differential diagnosis and consider the possibility of AAV, especially in cases of muscle weakness presenting as inflammatory myositis, in the absence of other clinical manifestations of systemic vasculitis or specific myositis serologies.

## Introduction

Antineutrophil cytoplasmic antibody (ANCA)-associated vasculitis (AAV), a small vessel vasculitis, presents with a wide spectrum of organ involvement and disease severity. The clinical symptoms could be from direct effects on organs or impeded blood flow to the end organs. Common clinical manifestations include constitutional symptoms such as weight loss, malaise, fever, myalgia, arthralgia, cutaneous lesions, rhinosinusitis, pulmonary infiltrates, glomerulonephritis, and mononeuritis multiplex. Although myalgia is common, true muscle weakness and inflammatory myopathy are rare manifestations of AAV [1]. Guillevin et al. reviewed 96 patients with eosinophilic granulomatosis with polyangiitis (EGPA) and noted that, while diffuse myalgia was present in 54% at presentation, concurrent myositis and loss of strength were not observed [1]. Similarly, Oiwa and Kurashige highlighted muscle weakness as a predominant presentation in only three cases of myeloperoxidase (MPO)-positive ANCA described in the article [2]. This case discusses a patient presenting primarily with features of myositis (including muscle weakness) in the setting of MPO-positive AAV. A literature review shows that myositis presenting as the predominant feature of AAV is rare.

## Case presentation

**Figure 1:**
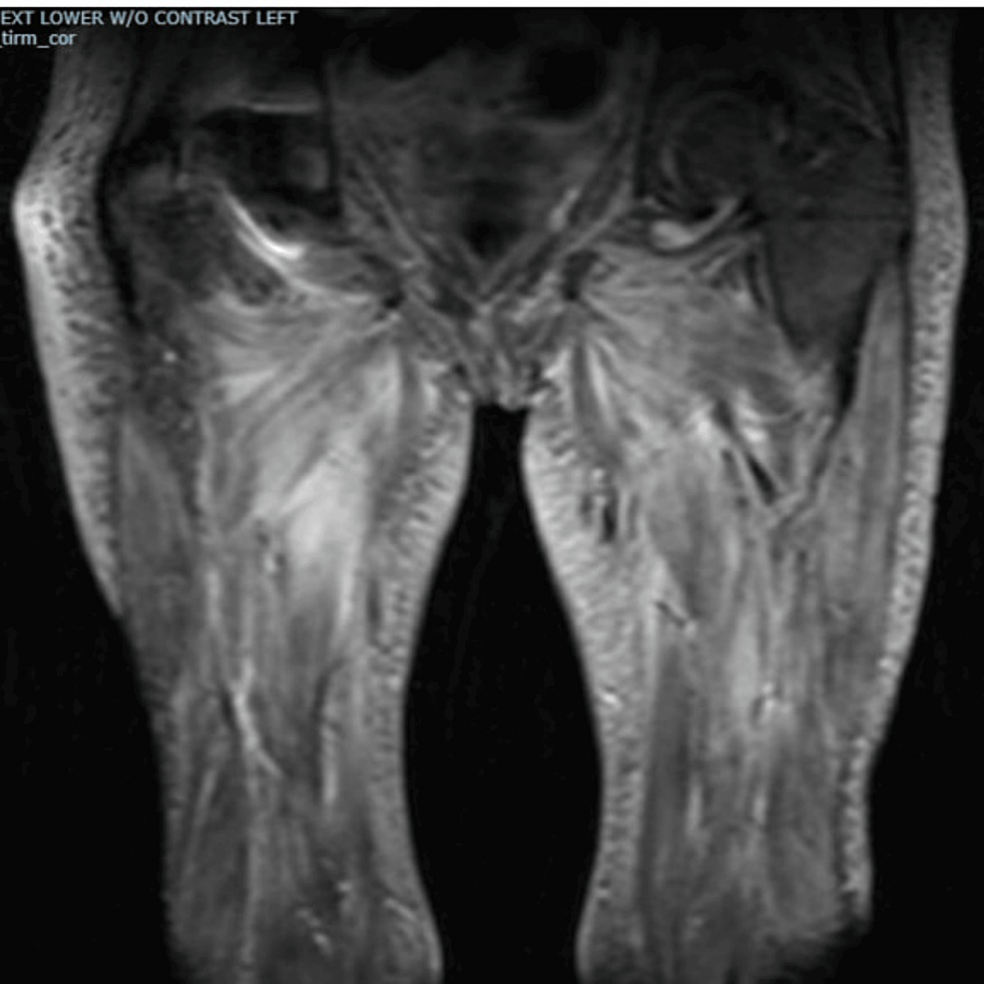
MRI of bilateral thighs showing muscle edema

A 77-year-old woman with hypothyroidism and osteoarthritis presented with a two-month history of worsening proximal muscle weakness, necessitating a walker and eventually a wheelchair. During the same period, she experienced dysphagia, a 40-lb unintentional weight loss, and persistent sinusitis with middle ear effusions, requiring bilateral tympanostomy. Physical examination revealed 2/5 muscle strength in hip flexors and extensors and 4/5 strength in other proximal muscle groups of the upper and lower extremities. Lower extremity MRI showed diffuse intramuscular edema between fat planes and intramuscular septal regions (Figure 1).

A complete blood count revealed normocytic anemia (Hgb 8.4 g/dL), an elevated platelet count (450,000 cells/microliter), and mild peripheral eosinophilia (0.65 K/uL; normal range: 0.04-0.36 K/uL). Additional findings included elevated erythrocyte sedimentation rate (70 mm/hr; normal <30), C-reactive protein (141 mg/L; normal <5), and creatine kinase (690 U/L; normal range: 29-168). Anti-MPO antibodies were markedly elevated (>999 AU/mL). Tests for antinuclear and myositis-specific autoantibodies, including anti-Jo-1, PL-7, PL-12, EJ-, OJ, SRP, Mi-2, MDA-5, NXP-2, SAE-1, PM/Scl-100, SS-A 52kD, U1-RNP, U2-RNP, U3-RNP (fibrillarin), and hydroxymethylglutaryl-CoA reductase antibodies, were negative. Thyroid studies were normal (Table 1).

**Table 1:** Laboratory findings

Laboratory tests	Result	Reference range
Hemoglobin (g/dL)	8.4	12-16
Platelets (cell/microliter)	450,000	150,000-450,000
Absolute eosinophils (K/uL)	0.65	0.04-0.36
Thyroid-stimulating hormone (ml/UL)	1.5	0.5-5.0
Alanine aminotransferase (IU/L)	26	0.0-44.0
Aspartate aminotransferase (IU/L)	25	0.0-40.0
Calcium (mg/dL)	9.5	8.6-10.4
Blood urea nitrogen (mg/dL)	8	6.0-20.0
Creatinine (mg/dL)	0.5	0.0-0.7
Potassium (mEq/L)	4.1	3.5-5.2
Magnesium (mg/dL)	1.9	1.6-2.3
Phosphorus (mg/dL)	3.2	2.8-4.1
Erythrocyte sedimentation rate (mm/hr)	70	0.0-30.0
C-reactive protein (mg/L)	141	0.0-5.0
Creatine kinase (U/L)	690	29-168
Anti-myeloperoxidase antibodies (AU/mL)	>999	<19
Antinuclear antibody	Negative	<1:40
Hydroxymethylglutaryl-CoA reductase antibody	Negative	-
Myositis-specific autoantibodies profile	Negative	-

**Figure 2:**
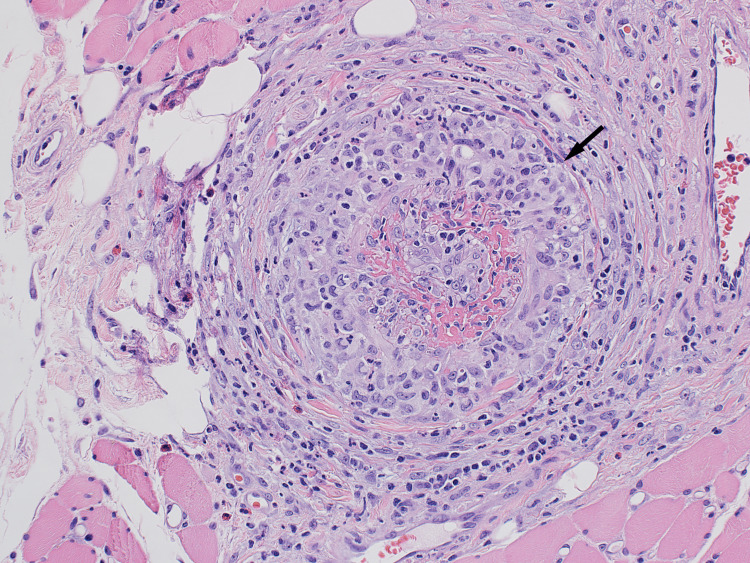
Histopathology slide of the thigh muscle biopsy

A thigh muscle biopsy revealed fibrinoid necrosis of small intramuscular arteries, confluent circumferential granulomatous vessel wall inflammation, and associated mild chronic inflammation, including occasional eosinophils and a few plasma cells (Figure 2).

**Figure 3:**
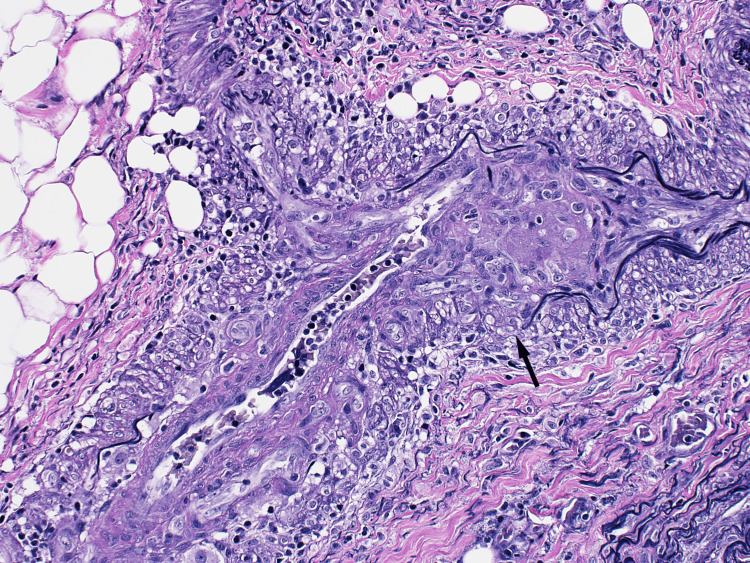
Histopathology slide of the thigh muscle biopsy

Elastin staining revealed segmental loss of small artery elastin fibers (Figure 3). 

Ultimately, the patient was diagnosed with myositis secondary to MPO-positive AAV. Treatment was started with oral prednisone at a 1 mg/kg daily dose and azathioprine (AZA) as a disease-modifying agent. After four months of treatment with AZA and tapering doses of prednisone, her weakness completely resolved, with 5/5 strength in bilateral upper and lower extremities. Sixteen months after the initial diagnosis, she was off prednisone, maintained on AZA therapy, and resumed previous levels of activity. A repeat MRI showed a complete resolution of muscle inflammation.

## Discussion

This patient’s clinical presentation of predominant lower extremity weakness due to inflammatory myositis is an unusual manifestation of AAV, including granulomatosis with polyangiitis (GPA), microscopic polyangiitis (MPA), and EGPA. Guillevin et al. reviewed 96 patients with EGPA and noted that, while diffuse myalgia was present in 54% at presentation, concurrent myositis and loss of strength were not observed [1]. Conticini et al. reviewed 395 patients with muscle vasculitis across 108 articles. They found that AAV accounted for approximately 25% of cases, with GPA representing 19%, MPA the most common subtype of AAV at 15%, and both GPA and EGPA each at 5% [3].

Our patient shared some of the above characteristics described in the literature. She was elderly and presented with lower extremity weakness and other systemic symptoms, including weight loss and fatigue. Her laboratory findings showed elevated inflammatory markers, moderately elevated creatine kinase, and high titer MPO antibody levels. Similarly, Oiwa and Kurashige highlighted muscle weakness as a predominant presentation in only three cases of MPO-positive ANCA described in the article [2]. Age and sex distribution are similar to those of AAV overall. In patients with myositis secondary to ANCA vasculitis, inflammatory markers may be elevated, but levels of CPK tend to be mild to moderate compared to idiopathic inflammatory myosis, as shown in Conticini et al.’s article. In this same review article, compared to PR3, MPO was more prevalent in patients who had inflammatory myositis on muscle biopsy [3]. MRI showed STIR and T2-weighted enhancement [3,4]. MRI findings in AAV myositis, although sensitive for detecting myofascial and fat plane edema, are nonspecific in appearance or very similar to myositis due to other etiologies. However, the study by Kawaguchi et al. suggested that fascial and subcutaneous fat hyperintensities may differentiate MPA-associated myositis from polymyositis or dermatomyositis [4]. Histopathology plays a vital role in the diagnosis of inflammatory myositis, but even more so in cases where the etiology might be unusual. Hervier et al.’s review of 310 muscle biopsies performed in one center between 2000 and 2008 found a final diagnosis of systemic vasculitis in 31 (10%). Twenty-two of those 31 cases (71%) were ANCA positive, while nine cases (29%) were ANCA negative [5,6].

There is no guideline for the treatment of isolated systemic vasculitis-associated myositis. However, the literature review showed patients were initially treated with high-dose oral steroids (prednisone up to 1 mg/kg) or IV steroids and with disease-modifying agents, including conventional synthetic disease-modifying antirheumatic drugs (methotrexate, AZA, or mycophenolate), with good outcomes. The patient above was treated with high-dose steroids and AZA (disease-modifying agent) for induction and maintenance therapy. The patient clinically improved, and the steroid was successfully tapered off. AZA, or methotrexate, is considered the choice for remission maintenance, especially in patients with non-renal disease [7]. The WEGENT trial (n = 126) showed that AZA and methotrexate therapy have similar efficacy in remission maintenance [7]. Mycophenolate mofetil is reserved for patients who cannot tolerate either of them. In the IMPROVE trial, mycophenolate is less effective than AZA for the maintenance of remission [7]. Rituximab and cyclophosphamide were not indicated since the patient did not have any organ damage [7].

## Conclusions

Myositis due to AAV is a rare occurrence. In a large cohort, MPA was found to be more common than GPA and EGPA. Clinical findings may include myalgia, especially calf tenderness, and muscle weakness with lower extremity involvement. Histopathology findings were predominantly consistent with necrotizing, fibrinoid, and vasculitis. Granulomatous vasculitis and eosinophilic vasculitis features were less common. In patients with muscle weakness presenting with inflammatory myositis but negative specific myositis autoantibodies, clinicians should consider the possibility of AAV as a possible differential despite the absence of other clinical manifestations of systemic vasculitis.
